# Paradoxical Adipose Hyperplasia of Submental Region After Cryolipolysis Treated With Deep-Plane Neck Lift: A Case Report

**DOI:** 10.1093/asjof/ojaf008

**Published:** 2025-01-31

**Authors:** Heather M Zimmerman, J Simon Ivey

## Abstract

Cryolipolysis is a technique to reduce adipose tissue with localized cooling. Paradoxical adipose hyperplasia (PAH) is a known risk of this procedure and presents with a voluminous swelling in the treated region, generally occurring 2 to 4 months after. Most cases of PAH are noted in the umbilical region, and it has been infrequently noted in the submental region. The researchers of previous reports have noted liposuction or excision as proposed treatments. In this study, the authors report on a case of PAH after 2 sessions of cryolipolysis to the submental region, which was treated with deep-plane neck lift (DPNL) for total correction. Intraoperative removal of sub- and supraplatysmal fibrotic fat, glandular material, and muscle was utilized for optimal results. The patient had total correction of the deformity and maintained results at several months postoperatively. This case would have been poorly treated with liposuction because of the dense structural material and fibrotic subplatysmal fat noted intraoperatively. Patients undergoing cryolipolysis for adipose reduction of the submental region should be counseled on the risk of PAH, and DPNL should be evaluated further as the standard for correction of this condition.

**Level of Evidence: 5 (Therapeutic):**

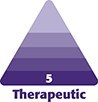

Cryolipolysis is an adipose reduction technique first cleared by the FDA for use in 2010. It uses localized cooling, proven selective for adipose cells.^1^ Thinning of the fat layer gradually occurs after treatment and has been demonstrated in both humans and animals.^1^ A topical applicator is applied (sometimes in multiple cycles and sessions), which cools and penetrates the fat layer, reducing the temperature to ∼0 °C.^2,3^ The effect of this cold exposure to the treated area culminates in adipose cell apoptosis, an effect preliminarily noted in female equestrians who had been riding in cold conditions and which remains poorly understood.^1^ The apoptosed adipose cells are then engulfed by infiltrating macrophages, a process that continues for up to 4 months after treatment.^1^

Although relatively free of long-term complications and effective, 1 undesirable risk of cryolipolysis is paradoxical adipose hyperplasia (PAH), which was first reported in a 2014 case report.^3^ A subsequent case series published in 2018 documented 1% of reviewed patients developing this complication in a variety of treated regions, including 1 case in the submental region.^4^

PAH is generally noted to appear between 2 and 4 months after treatment and presents as a painless swelling in the treated region, resembling increased, recurrent adiposity.^4^ The growth eventually stabilizes, is normally in the umbilical region, and previous authors have proposed liposuction for its removal; however, we report on a case of PAH after cryolipolysis in the submental region treated with a deep-plane neck lift (DPNL) for total correction.

## CASE REPORT

**Figure 1.**
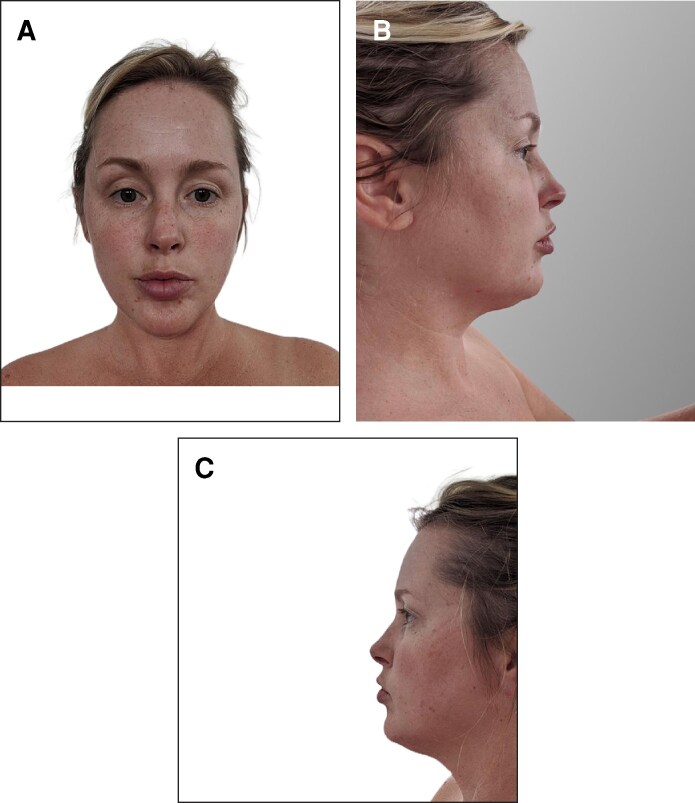
(A) Baseline image of a 39-year-old female demonstrating the appearance of the submental region before cryolipolysis. Frontal view: this photograph was provided by the patient. (B) Baseline image of a 39-year-old female demonstrating the appearance of the submental region before cryolipolysis. Right, lateral view: this photograph was provided by the patient. (C) Baseline image of a 39-year-old female demonstrating the appearance of the submental region before cryolipolysis. Left, lateral view: this photograph was provided by the patient.

**Figure 2.**
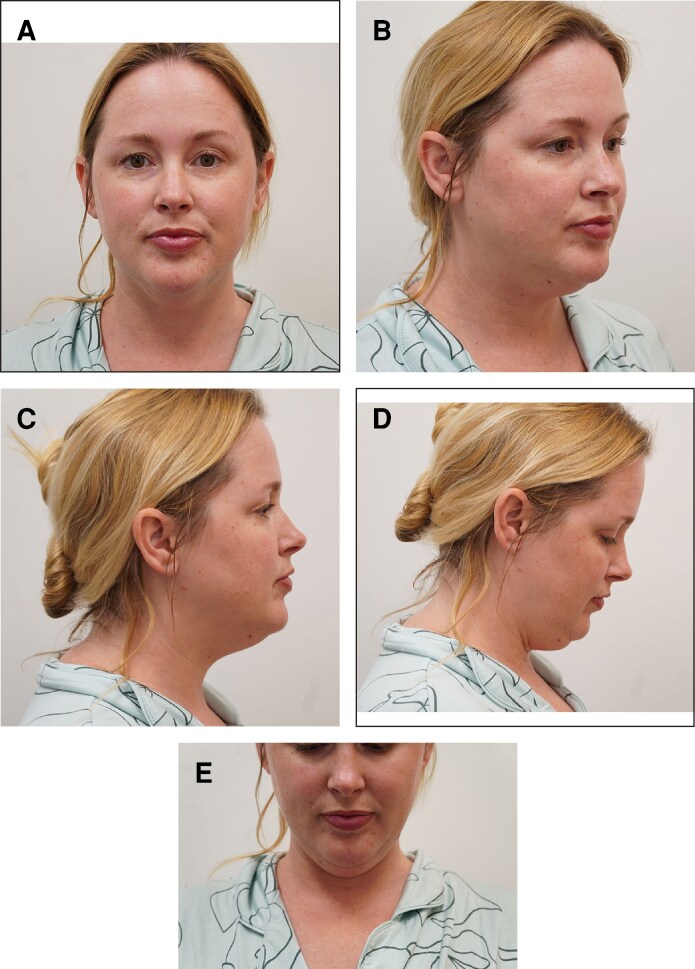
(A) Preoperative image of a 39-year-old female demonstrating a voluminous mass in the submental region after cryolipolysis (frontal view). (B) Preoperative image of a 39-year-old female demonstrating a voluminous mass in the submental region after cryolipolysis (3 quarters view). (C) Preoperative image of a 39-year-old female demonstrating a voluminous mass in the submental region after cryolipolysis (lateral view). (D) Preoperative image of a 39-year-old female demonstrating a voluminous mass in the submental region after cryolipolysis (lateral view, worse with anterior flexion). (E) Preoperative image of a 39-year-old female demonstrating a voluminous mass in the submental region after cryolipolysis (frontal view, worse with anterior flexion).

A 39-year-old white female presented, reporting increased adiposity in the submental region (Figures 1, 2). Medical history was insignificant aside from deoxycholic acid injection, followed by cryolipolysis. She received 2 treatments of cryolipolysis to the submental areas. The increased adiposity was reported to occur after cryolipolysis without any reported weight gain. Previous therapies attempted included weight reduction, with no relief. Physical examination revealed fullness in the submentum with slight firmness, consistent with PAH (Figure 2).

**Figure 3.**
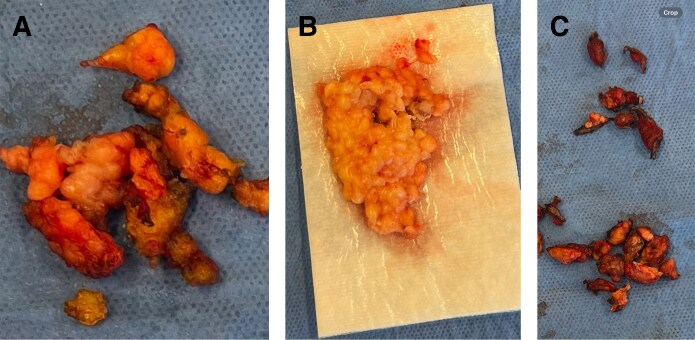
(A) Subplatysmal fibrotic adipose tissue consistent with paradoxical adipose hyperplasia. (B) Supraplatysmal fibrotic adipose tissue consistent with paradoxical adipose hyperplasia. (C) Portions of anterior digastric muscle and submandibular salivary gland were removed for additional neck definition.

**Figure 4.**
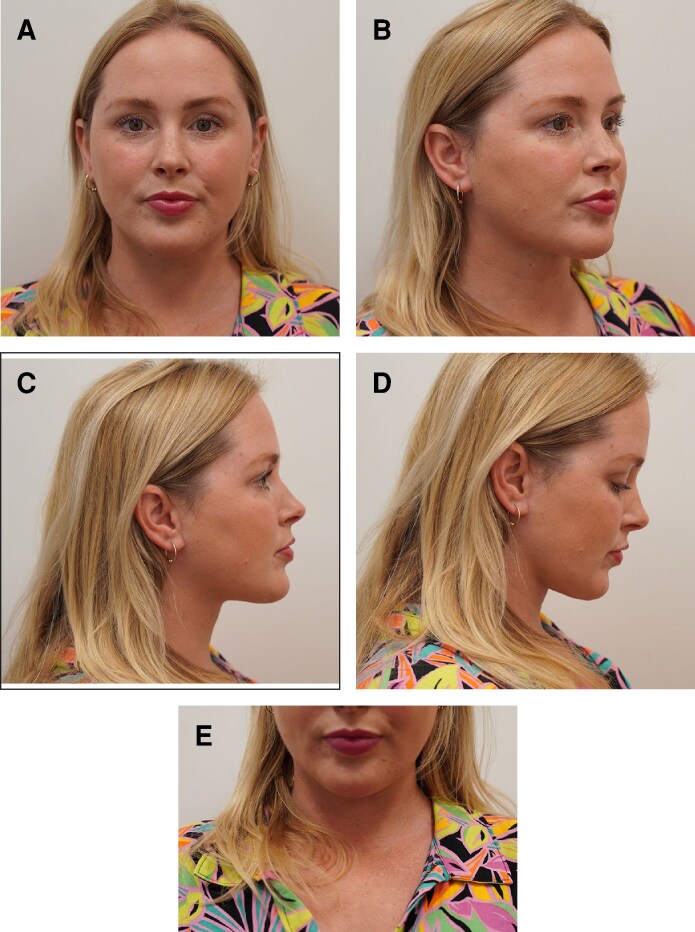
(A) Follow-up image of a 39-year-old female who was treated for paradoxical adipose hyperplasia (PAH). The results are maintained at 4 months postoperatively, after deep-plane neck lift (DPNL) and micro fat injection into the prejowl area (frontal view). (B) Follow-up image of a 39-year-old female who was treated for PAH. The results are maintained at 4 months postoperatively, after DPNL and micro fat injection into the prejowl area (3 quarters view). (C) Follow-up image of a 39-year-old female who was treated for PAH. Results are maintained at 4 months postoperatively, after DPNL and micro fat injection into the prejowl area (lateral view). (D) Follow-up image of a 39-year-old female who was treated for PAH. The results are maintained at 4 months postoperatively, after DPNL and micro fat injection into the prejowl area (lateral view, with anterior flexion). (E) Follow-up image of a 39-year-old female who was treated for PAH. Results are maintained at 4 months postoperatively, after DPNL and micro fat injection into the prejowl area (frontal view, with anterior flexion).

**Figure 5.**
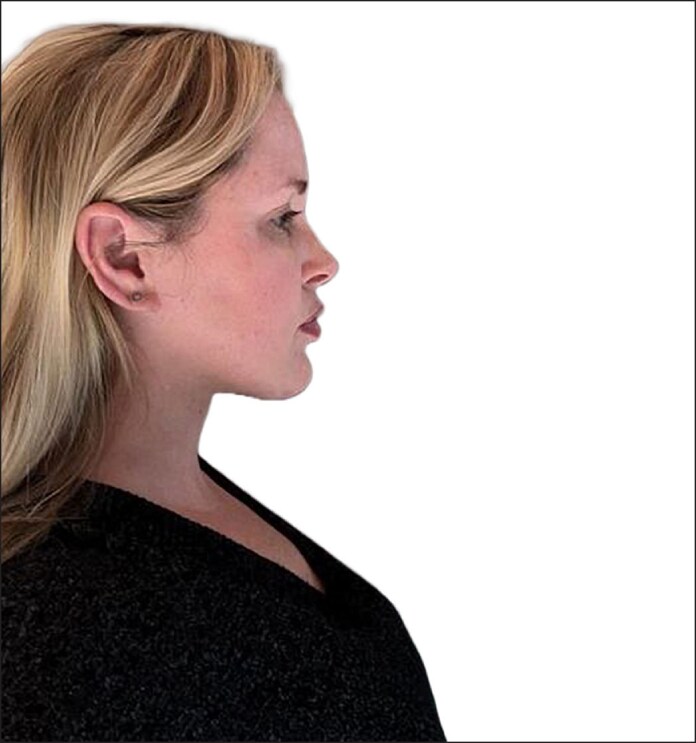
Follow-up image of a 39-year-old female who was treated for paradoxical adipose hyperplasia. The results are maintained at 7 months postoperatively, after deep-plane neck lift and micro fat injection into the prejowl area (lateral view). This photograph was provided by the patient.

The patient subsequently underwent an elective DPNL, as follows: The skin was first fully undermined in the submental area down to the thyroid leaving an appropriate flap thickness. Supraplatysmal fat was directly excised off the platysma. Then, a spatula lipo cannula was used to suction the rest of the platysma in an open fashion. The platysma was opened and raised as flaps from the midline. The subplatysmal fat was reduced. The anterior digastric was reduced bilaterally. The submandibular salivary gland capsule was opened and dissected and partially excised with a Ligasure exact device. Ten units of botulinum toxin were injected into each salivary gland stump under direct vision. The platysma was then sutured into the midline in an interrupted fashion. Laterally, postauricular flaps were raised with an incision below the tragus extending postauricular. The deep plane was entered in the masseteric area, and the masseteric retaining ligaments were released along with a full release of the platysma off the parotid capsule. Suspension of the lateral platysma was performed using the mastoid crevasse technique, after release of the cervical retaining ligaments.^5^ A small amount of skin was removed postauricularly. Approximately 75% of the removed fatty material was noted to be fibrotic, consistent with PAH (Figure 3). Micro fat injection into the prejowl area to further enhance her jawline was performed. The procedure was well tolerated, and recovery was swift, with maintained results at 4 and 7 months postoperative (Figures 4, 5).

Patient consent was obtained before image use. This study was conducted in accordance with the Declaration of Helsinki.

## DISCUSSION

Although PAH was a known issue after the introduction of cryolipolysis, with some 33 cases present at the time of the first published report, the manufacturers proposed that reduction in applicator size may mitigate this side effect.^4^ Even so, the results of a subsequent case series published in 2018 demonstrating PAH in 1% of patients seemed to disagree with applicator size as a contributor.^4^ In a future multicenter study in Canada, the authors revealed that 9 out of 2114 patients experienced the side effect itself, or 0.43%.^6^ Both studies cited risk factors of male sex, large applicator, and periumbilical region; however, these criteria were at total odds with this case report because the patient was female and treated in the submental region, presumably with an appropriately small applicator.^4,6^

Proposed corrections for PAH include liposuction, excision, or more extensive surgeries if skin laxity is involved; however, this approach discounts the possibility that subplatysmal fat could also be affected by PAH.^4,6,7^

In this report, the authors further demonstrate that patients opting for nonsurgical treatments, such as cryolipolysis, to reduce adiposity of the submental region are at risk of PAH in both the sub- and supraplatysmal spaces. Patients should be informed of the risk of PAH in the submental region after cryolipolysis, and DPNL of the submental region should be investigated further as the standard for correction, because the subplatysmal PAH cannot be treated with liposuction. Moreover, patients should be afforded the best possible outcome after enduring such a detrimental cosmetic consequence. Limitations included a lack of information about previous treatments, including the amount of deoxycholic acid injected and the precise number of cryolipolysis cycles.

## CONCLUSIONS

This case highlights an additional occurrence of the sparsely reported manifestation of PAH in the submental region, with full surgical correction through DPNL.^4,7^ We contend that utilization of cryolipolysis for this region should be closely monitored for PAH in future practice, and patients should be advised of potential risk. A return to baseline may not be feasible with liposuction and/or excision alone because of skin laxity and subplatysmal contribution, so further studies should explore DPNL as a treatment for submental PAH to improve outcomes for these patients.
